# The effect of group size and laying month on the quality, IgG, and corticosterone levels of goose eggs

**DOI:** 10.5194/aab-67-551-2024

**Published:** 2024-11-19

**Authors:** Lili Dóra Brassó, István Komlósi, Levente Czeglédi, Gabriella Gulyás

**Affiliations:** 1 Department of Animal Science, Institute of Animal Science, Biotechnology and Nature Conservation, Faculty of Agricultural and Food Sciences and Environmental Management University of Debrecen, 138 Boszormenyi Street, 4032 Debrecen, Hungary

## Abstract

Environmental stress is known to negatively affect poultry health, production, and egg quality. Our study aimed to evaluate the effects of two different group sizes and the laying month on egg quality characteristics as well as the effect of group size on corticosterone and IgG levels in goose eggs. The research was conducted on a semi-free-range goose breeder farm in Hajdú–Bihar county, Hungary. The eggs included in the analysis were produced by 4-year-old geese of the Grimaud breed. Two group sizes were constructed; the large group contained 850 birds; and there were three small groups, each containing 50 geese as replicates. The effect of the laying month and group size on egg quality parameters and the effect of group size on egg IgG and corticosterone contents were investigated. Eggs laid in January at the peak of production and at the end of February (low-production period) were involved in the study. Regarding the effect of months, we noticed a decrease in egg width (from 6.12 to 5.98 cm ), shell thickness (from 0.76 to 0.61 mm at the blunt end, from 0.69 to 0.61 mm at the equator, and from 0.65 to 0.56 mm at the pointed end), shell weight (from 19.56 to 18.19 g ), yolk weight (from 69.05 to 62.35 g ), yolk ratio (from 36.45 % to 34.43 %), yolk diameter (from 7.09 to 6.59 cm ), and yolk colour with fan (from 12.58 to 11.83) and $b^{*}$ (from 54.57 to 49.91) ( P ≤ 0.05). The albumen ratio and yolk pH increased from 53.24 % to 55.51 % and from 6.18 to 6.29 from January to February, respectively. Regarding group size, the albumen pH (8.77 vs. 8.67), IgG (4955 vs. 3823 $\mathrm{ng}\;{\mathrm{mL}}^{-1}$ ), and corticosterone (187.26 vs. 76.24 $\mathrm{ng}\;{\mathrm{mL}}^{-1}$ ) levels were higher in the small groups ( P ≤ 0.05).

## 1 Introduction

Goose egg, besides chicken, quail, and duck eggs, is a cheap food source with high nutrient content (Wijedasa et al., 2020). Egg quality and characteristics (i.e. egg weight, egg content weight, shell thickness, etc.) have a great impact on embryonic development and gosling hatchability (Narushin and Romanov, 2002; Biesiada-Drzazga et al., 2016). For example, Mitrovic et al. (2018) established correlations between goose egg shape index and weight, egg hatchability, and weight of goslings. Egg weight and gosling weight had a strong positive correlation ( r = 0.986), and they revealed a weak positive correlation between egg shape index and egg weight ( r = 0.180). Dead-in-shell eggs were the heaviest (175.92 g ), and infertile eggs had the lowest weights (167.08 g ) (Mitrovic et al., 2018). Being aware of these connections between egg quality and hatchability, the analysis of egg parameters is crucial. Besides genetic and nutritional effects (Sun et al., 2019), egg quality is also influenced by seasonal effects. In the case of 2-year-old or older birds, the weight of goose eggs decreases during the laying season. Lighter eggs can result in smaller goslings and affect their quality (Brun et al., 2003; Bogenfürst, 2017). The shell weight, shell thickness, yolk weight, albumen height, Haugh unit, and the area of thick albumen also show a decrease over the laying season (Razmaite et al., 2014; Mazanowski and Adamski, 2006; Dodu, 2010).

Regarding environmental conditions, stress poses negative effects on poultry health and performance and causes alterations in egg quality parameters (Alagawany et al., 2017). Higher-than-normal stocking density and group sizes (especially in poultry and pigs; Rodenburg and Koene, 2007), heat stress (Gui-Ming et al., 2020), feed restriction, sudden environmental changes (Nicol et al., 2006), and diseases can be stressful for the birds (Thaxton et al., 2006; Delezie et al., 2007; Kang et al., 2016). For poultry, the group size found in the wild is generally smaller than the group size applied in farm conditions. Large group sizes may lead to problematic and aggressive behaviour and increased fear and stress levels (Rodenburg and Koene, 2007; Bilcík and Keeling, 2000). Although a few studies have described low levels of aggression at large group sizes in poultry (Nicol et al., 1999; Estevez et al., 2002; Estevez et al., 2003), the traditional model suggests that aggression increases with increasing group size (Estevez et al., 2007). The elevated levels of aggression result in a decline in egg fertility and hatchability (Mench, 1993).

Due to stress, the HPA (hypothalamic–pituitary–adrenal) axis becomes activated and stimulates the release of glucocorticoids from the adrenal gland (Carsia and Harvey, 2000). Corticosterone is one of the main avian glucocorticoid stress hormones triggering gluconeogenesis (Carsia and Harvey, 2000). Several functions of corticosterone have been described: it takes part in the regulation of fuel metabolism, feed intake, and immune responses. Everyday corticosterone administration into the body of broilers results in a higher water and feed intake, reduced growth, and pronounced gluconeogenesis and proteolysis (Siegel and Van Kampen, 1984; Lin et al., 2004). Stress results in higher corticosterone concentration levels in blood plasma (Henriksen et al., 2011) and eggs (Saino et al., 2005), reducing egg fertility and hatchability (Schmidt et al., 2009). The increase in the corticosterone concentration in blood plasma also negatively affects the function of the immune system, delays the onset of the laying season, and reduces persistence and egg production (Shini et al., 2008). Besides the previously mentioned effects, corticosterone negatively impacts embryo development and egg quality parameters (Kim et al., 2015; Downing and Bryden, 2008). In hens, corticosterone supplementation of feed increases eggshell thickness and yolk redness and decreases the Haugh unit (Kim et al., 2015). There is increasing interest in using non-blood, non-invasive measures to assess stress in farm animals. The egg can be used as a non-invasive biological sample for corticosterone quantification. Indeed, 80 % of corticosterone can be found in the yolk, and 20 % is stored in the albumen (Royo et al., 2008).

In birds, there are three types of immunoglobulins: IgA, IgM, and IgY. IgM and IgA are similar to that of mammals, IgY is equal to the mammalian IgG (Carlander et al., 1999). IgM and IgA are present in the albumen, while the yolk is rich in IgY (Vaillant and Ferrer-Cosme, 2021). In poultry, the mother can pass immunoglobulins to the offspring through the egg and provide passive immunity for the offspring. In the literature, there are conflicting results on the relationship between stress and plasma IgG levels. Stress factors (Nicol et al., 2006) can mitigate the immune response by inhibiting the growth of immune organs (Liu et al., 2014), resulting in the reduction of immune cells such as immunoglobulins (Roushdy et al., 2020). On the contrary, chronic heat stress increases IgG concentration in the plasma of hens (Li et al., 2020). Yang et al. (2015) concluded that the corticosterone administration resulted in higher plasma IgG levels in broiler chickens. The effect of group size on the quality, IgG, and corticosterone levels of goose eggs has not been analysed yet.

Our study aimed to evaluate the effects of two different group sizes (large group of 850 geese vs. small groups of 50 geese per group) and the laying month (at the peak of production in January and at the end of production in February) on egg characteristics and the effect of group sizes on the corticosterone and IgG levels of goose eggs. We can prove by the analyses whether the group size or laying month affects egg quality, goose immunity, and stress level. Our findings can improve animal welfare via the establishment of an optimal husbandry system (large group vs. small group).

## 2 Materials and methods

### 2.1 Study population and experimental design

The eggs included in the analyses were laid by 4-year-old geese of a Grimaud goose parent stock (Tranzit-Ker Ltd., Érpatak, Hungary), between December 2020 and February 2021. The Grimaud is a French white-coloured meat-purpose hybrid. A barn (10 m × 100 m in size) containing 1000 birds with 1:4 ( gander:goose ) sex ratio and 1 bird per $m^{2}$ population density was involved in the experiment. At the beginning of November, right after the standard date of housing, one part (15 m × 10 m ) of the barn was isolated, and three small groups, each 50 $m^{2}$ in size (10 m × 5 m ), were constructed. Geese in the small groups were randomly selected from the original population, maintaining sex ratio ( 1 gander:4 geese ) and population density (1 bird per $m^{2}$ ). The three small groups contained 150 geese (50 birdseach ) altogether, and the population size was 850 birds in the large group (1 bird per $m^{2}$ ). Groups were separated with wooden fences that enabled communication between them. Flock sizes in small groups (50 birdspergroup ) were determined according to the standard population size (10–60 birdseach ) of migrating wild geese (Lengyel et al., 2012) and also from a management and statistical point of view to draw reliable conclusions. The size of the large group (850 birds) was typical of flock sizes in the barns on Hungarian farms. The laying season started at the onset of January (Brassó et al., 2024), so birds had 8 weeks to adapt to the new environment until the start of egg production.

**Table 1:** Summary of the evaluated egg quality parameters.

External egg quality parameters	Internal egg quality parameters
Egg weight ( g )	Albumen weight ( g )
Egg length ( cm )	Albumen ratio (%)
Egg width ( cm )	Yolk weight ( g )
Egg shape index (%)	Yolk ratio (%)
Egg volume ( ${\mathrm{cm}}^{3}$ )	Yolk diameter ( cm )
Egg specific gravity ( $g\;{\mathrm{cm}}^{-3}$ )	Yolk colour ( $L^{*}$ , $a^{*}$ , $b^{*}$ )
Weight of eggshell with membranes ( g )	Yolk pH
Shell thickness ( mm )	Albumen pH
Shell surface ( ${\mathrm{mm}}^{2}$ )	
Shell density ( $g\;{\mathrm{cm}}^{-3}$ )	
Shell weight ( g )	
Shell ratio (%)	

### 2.2 Feeding and husbandry technology

The feeding and husbandry technology was the same in all groups. The barn was deeply bedded with straw. Birds had permanent access to feed and fresh water. From June until the first half of November, the geese dwelled on the pasture, consumed 180 g per bird per day concentrate diet for maintenance, and had ad libitum access to straw and hay. The birds were reared in a semi-free-range system, including a 1000 $m^{2}$ stable and a 700 $m^{2}$ paddock. From the second half of November, they received 200 g of a goose layer diet, and then the ratio was raised by 50 g per week for another 2 weeks. From the onset of December, geese were given an ad libitum (350 g ) layer's diet until the point when egg production fell below 40 %; after that, a 300 g per bird per day layer's diet was provided. During the laying period, the feed was supplemented with gravel and microelements dissolved in the drinking water. The lightning programme was started at the end of November, with neon and LED lights with 40–60 lx light intensity lifting the light period every week by 0.5–1 h , reaching a maximum of 13 h . From this point, egg production was ongoing in natural light.

### 2.3 Collection and evaluation of eggs

Altogether, 40 eggs were collected for egg quality analysis in the second half of January (at the peak of production; 60 % of egg production when 60 % of the geese laid eggs each day) and at the end of February (at the end of the laying season; laying intensity decreased to below 40 %). In January, 10 of the 40 eggs stemmed from the large group and 10 were laid in each small group (30 altogether). In February, 10 eggs were collected from the small groups per replicate (30 altogether) and 10 originated from the large group. In February, eight eggs were randomly chosen from groups for both IgG and corticosterone analysis. The following external and internal characteristics (Sari et al., 2019) were analysed for the egg quality evaluation (Table 1).

Eggs were refrigerated right after collection and stored at 4 °C for 1 d before the egg quality analysis. A three-decimal digital analytical balance was used for the measurement of egg weight (VWRI611-2263; Italy, 2020). The determination of egg length and width was carried out with a two-decimal calliper. The shape index was expressed as the quotient of egg width and length in percentages. A measuring pot filled with water and placed on a three-decimal digital balance (VWRI611-2263; Italy, 2020) was used for the identification of egg volume. The eggs were immersed in water in a wire basket without touching the bottom and/or the wall of the pot. The weight of the pot, water, and wire basket was weighed after egg weight measurement to avoid distorting the results. The specific gravity was calculated by the quotient of egg weight and egg volume. Egg yolks and albumens for the measurements were separated very carefully to prevent contamination. The unused parts of the yolk samples were stored in cryotubes at - 80 °C until molecular analyses. Eggshell thickness was measured with a two-decimal calliper at three points: at the equator, at the pointed end, and at the blunt end with three replicates. Eggshell surface and eggshell density were calculated by the formulae 3.9782^0.7056^ and 1.945^0.014^, respectively (Nedomová and Buchar, 2014). The weight of the dried eggshell and the egg yolk were weighed with a three-decimal digital balance. The weight of the albumen was calculated using the difference between the eggshell and yolk weight. The ratio of egg components was expressed in percentages. The egg yolk diameter was established with a two-decimal calliper. A yolk fan and a Konica Minolta CR-410 Chroma Meter were used for the determination of egg yolk colour. The egg yolk and albumen pH were measured with a Testo AG Germany 205 pH gauge.

**Table 2:** Changes in the main external characteristics of goose eggs in the evaluated laying months and groups (mean ± standard error of the mean, SEM).

Month/parameter	Egg weight	Egg length	Egg width	Shape index	Egg volume	Egg specific gravity
	( g )	( cm )	( cm )	(%)	( ${\mathrm{cm}}^{3}$ )	( $g\;{\mathrm{cm}}^{-3}$ )
Jan ( n = 40)	189.63 ± 4.94	9.09 ± 0.09	6.12 ± 0.04^b^	67.38 ± 0.65	174.71 ± 3.15	1.09 ± 0.01
Feb ( n = 40)	181.24 ± 4.94	9.03 ± 0.09	5.98 ± 0.04^a^	66.36 ± 0.65	166.74 ± 3.15	1.09 ± 0.01
Large group ( n = 20)	185.79 ± 4.65	9.13 ± 0.09	6.08 ± 0.04	67.64 ± 0.66	174.24 ± 3.06	1.09 ± 0.01
Small groups ( n = 60)	185.07 ± 4.65	9.03 ± 0.09	6.03 ± 0.04	66.09 ± 0.66	170.21 ± 3.25	1.09 ± 0.01
Large group in Feb ( n = 10)	181.79 ± 4.75	8.97 ± 0.14	6.00 ± 0.05	67.06 ± 1.13	167.31 ± 4.36	1.09 ± 0.01
Small groups in Feb ( n = 30)	182.42 ± 2.91	8.96 ± 0.09	6.03 ± 0.03	67.43 ± 0.69	167.95 ± 2.67	1.09 ± 0.01

^a, b^ Letters indicate significant differences ( P ≤ 0.05).

### 2.4 Egg sample preparation and enzyme-linked immunosorbent assay for IgG and corticosterone analysis

The yolk samples were thawed at room temperature, and 300 mg from samples was transferred to sterile tubes containing 750 µl cold phosphate-buffered saline (1xPBS, pH 7.4) and homogenized with ULTRA-TURRAX. The mixtures were centrifuged at 4000 g for 10 min at 4 °C . The aqueous fractions were collected and centrifuged twice more for a total of three cycles. The aqueous supernatants were used to quantify IgG and corticosterone with enzyme-linked immunosorbent assays (ELISA). A commercial chicken Immunoglobulin G (IgG) double-sandwich ELISA Kit (Xinqidi Biotech Co., Ltd., Wuhan, China) was applied to determine the yolk IgG concentration and a commercial chicken CORT (corticosterone) competitive ELISA Kit (Wuhan Fine Biotech Co., Ltd., Wuhan, China) was used to determine the yolk corticosterone concentration. Assays were performed according to the manufacturer's instructions. Yolk samples were diluted 25 times for IgG determination, and the aqueous supernatants were utilized directly for corticosterone determination. All samples and standards were measured in duplicate. Absorbance data were collected with a Synergy HTX Multi-Mode microplate reader (Agilent, CA, United States) set to 450 nm . Sample concentrations were interpolated from a standard curve using BioTek Gen5 data analysis software (Agilent, CA, United States). The sensitivity of the assays for IgG and corticosterone was 5 and 1.688 $\mathrm{ng}\;{\mathrm{mL}}^{-1}$ , respectively. The intra-assay coefficient of variation for IgG and corticosterone was < 10 %.

### 2.5 Statistical analysis

Statistical differences between egg characteristics by group sizes and laying months were calculated with the IBM SPSS Statistics 23.0 program. Multivariate analysis of variance and Tukey's test with a significance level of P ≤ 0.05 were used for the comparison of mean values. The effect of laying month and group size was investigated as the main effect and also in interaction. Egg quality characteristics, IgG, and corticosterone concentrations were dependent variables, while group sizes and laying months were fixed factors. Values in the small groups are indicated as the mean of the three small groups.

## 3 Results and discussion

The interaction of laying month and group size did not affect any of the analysed characteristics of goose eggs ( P = 0.11 to 0.98). So, results are presented for months and groups as fixed main effects. In our study, we aimed to evaluate the effect of laying month and group size on egg quality and the effect of group size on egg corticosterone and IgG levels.

### 3.1 The effect of laying month and group size on egg external and internal quality parameters

Regarding external characteristics, only the egg width ( P = 0.016) decreased from January to February (Table 2). However, there was no change in the external parameters between groups. Although there was an 8 g difference in the egg weights and an 8 ${\mathrm{cm}}^{3}$ difference in the egg volume between months, the changes were not significant (egg weight: P = 0.09; egg volume: P = 0.053). The grand means of the examined external parameters were the following: egg weight was 185.44 g , length 9.06 cm , width 6.05 cm , shape index 66.87 %, egg volume 170.73 ${\mathrm{cm}}^{3}$ , and specific gravity 1.09 $g\;{\mathrm{cm}}^{-3}$ . External egg characteristics did not differ by groups in February ( P = 0.72 to 0.96).

**Table 3:** Changes in the quality parameters of goose eggshell in the evaluated laying months and groups (mean ± SEM).

Month/parameter	Shell thickness at the	Shell thickness at the	Shell thickness at the	Shell surface	Shell density
	pointed end ( mm )	equator ( mm )	blunt end ( mm )	( ${\mathrm{mm}}^{2}$ )	( $g\;{\mathrm{cm}}^{-3}$ )
Jan ( n = 40)	0.76 ± 0.02^b^	0.69 ± 0.02^b^	0.65 ± 0.02^b^	160.97 ± 2.04	2.028 ± 0.01
Feb ( n = 40)	0.61 ± 0.02^a^	0.61 ± 0.02^a^	0.56 ± 0.02^a^	155.92 ± 2.04	2.026 ± 0.01
Large group ( n = 20)	0.69 ± 0.02	0.65 ± 0.02	0.59 ± 0.02	158.66 ± 1.98	2.03 ± 0.01
Small groups ( n = 60)	0.68 ± 0.02	0.66 ± 0.02	0.62 ± 0.02	158.24 ± 2.21	2.03 ± 0.01
Large group in Feb ( n = 10)	0.62 ± 0.01	0.59 ± 0.02	0.56 ± 0.02	156.26 ± 2.88	2.03 ± 0.01
Small groups in Feb ( n = 30)	0.59 ± 0.01	0.60 ± 0.01	0.55 ± 0.01	156.61 ± 1.77	2.03 ± 0.01

^a, b^ Letters indicate significant differences ( P ≤ 0.05).

**Table 4:** Changes in the ratio of the main egg components in the evaluated laying months and groups (mean ± SEM).

Month/parameter	Shell weight	Albumen weight	Yolk weight	Shell ratio	Albumen ratio	Yolk ratio
	( g )	( g )	( g )	(%)	(%)	(%)
Jan ( n = 40)	19.56 ± 0.47^b^	101.02 ± 2.32	69.05 ± 1.33^b^	10.31 ± 0.20	53.24 ± 0.53^a^	36.45 ± 0.51^b^
Feb ( n = 40)	18.19 ± 0.47^a^	100.71 ± 2.32	62.35 ± 1.33^a^	10.06 ± 0.20	55.51 ± 0.53^b^	34.43 ± 0.51^a^
Large group ( n = 20)	18.73 ± 0.48	100.21 ± 2.49	66.87 ± 1.53	10.08 ± 0.19	53.91 ± 0.52	36.01 ± 0.49
Small groups ( n = 60)	19.02 ± 0.48	101.52 ± 2.49	64.53 ± 1.53	10.29 ± 0.21	54.84 ± 0.55	34.87 ± 0.53
Large group in Feb ( n = 10)	17.90 ± 0.69	100.85 ± 3.32	63.04 ± 1.68	9.86 ± 0.29	55.47 ± 0.72	34.67 ± 0.70
Small groups in Feb ( n = 30)	17.96 ± 0.43	102.32 ± 2.03	62.14 ± 1.03	9.85 ± 0.18	56.02 ± 0.44	34.13 ± 0.43

^a, b^ Letters indicate significant differences ( P ≤ 0.05).

Nedomová and Buchar (2014), studying 3-year-old Landes geese, established the mean egg weight to be 163.69 g , which is 23 g lower than the Grimaud eggs in our experiment. Saatci et al. (2005) claimed that the mean egg weight and shape index were 148.43 g and 66.80 %, deriving from a 4-year-old Turkish goose population. Zhang et al. (2017) examined a 2-year-old flock of unknown genotype and found the mean goose egg length, width, and shape index to be 7.826 mm , 5.362 mm , and 69 %. In comparison with the study of Zhang et al. (2017), the eggs in our examination were larger but more ellipsoid. Nedomová and Buchar (2014) stated that the mean goose egg length, width, and shape index of Landes geese were 8.96 cm , 5.816 cm , and 65.03 %. The fact that we had bigger eggs can be accounted for by the older flock or the different genotype we examined. The mean egg volume and specific gravity were 171.27 ${\mathrm{cm}}^{3}$ and 1.09 $g\;{\mathrm{cm}}^{-3}$ . The egg volume was 10 ${\mathrm{mm}}^{3}$ lower in February compared to January. Nedomová and Buchar (2014) determined the mean egg volume to be 159.21 ${\mathrm{cm}}^{3}$ . Karabulut (2021) found egg specific gravity of grey Chinese, Linda, and Aksaray native goose breeds to be 1.09 ± 0.01 ${\mathrm{cm}}^{3}$ , which is in correlation with our results.

The mean shell thickness showed the lowest value at the blunt end and increased from the blunt to the pointed end (Table 3). Group size did not have an impact on the shell parameters. The grand means of the examined shell characteristics were the following: shell thickness at the pointed end was ,0.69 mm , shell thickness at the equator 0.65 mm , shell thickness at the blunt end 0.61 mm , shell surface 158.44 ${\mathrm{mm}}^{2}$ , and shell density 2.03 $g\;{\mathrm{cm}}^{-3}$ . Eggshell characteristics did not differ by groups in February ( P = 0.25 to 0.99). Shell thickness was the thickest at the pointed end and showed a decreasing tendency to the blunt end, which is in agreement with the findings of Bogenfürst (2017). Apart from shell thickness, other shell characteristics did not show changes in the examined period.

Adamski et al. (2016) investigated Biała Kołudzka geese and declared that in the third laying season, the mean eggshell thickness was 0.566 mm . Biesiada-Drzazga et al. (2016) claimed that the mean eggshell thickness was 0.528 mm for Koluda geese of the same age. Results from the authors showed lower mean eggshell thickness. Nedomová and Buchar (2014) stated that the mean eggshell surface of Landes geese was 159.206 ${\mathrm{mm}}^{2}$ , 0.43 ${\mathrm{mm}}^{2}$ larger than the value we measured. Adamski et al. (2016) found the eggshell density of Biała Kołudzka geese to be 2.13 ± 0.17 $g\;{\mathrm{cm}}^{-3}$ , which is slightly greater than in our study. The effect of group size and laying month on shell characteristics is not available in the literature.

The albumen made the largest part of the egg components, weighing 101 g and amounting to 54 % (Table 4). The yolk made up one-third of the egg weight, and the shell was 1/10 of it. The egg components did not differ by group. However, we could demonstrate a decrease in the shell weight, yolk weight, and yolk ratio from January to February. At the same time, the albumen ratio increased due to the changes in the other two egg components. The grand means of the examined egg components were the following: shell weight was 18.88 g , albumen weight was 100.86 g , yolk weight 65.70 g , shell ratio 10.18 %, albumen ratio 54.38 %, and yolk ratio 35.44 %. The ratio of the main egg components did not differ by groups in February ( P = 0.52 to 0.97).

Biesiada-Drzazga et al. (2016) examined eggs of 4-year-old Koluda white geese and found that the weight of yolk, albumen, and shell was 59.6, 105, and 26.52 g . References including the changes of egg components by laying month and group size are not available.

**Table 5:** Changes in the yolk characteristics and albumen pH in the evaluated laying months and groups (mean ± SEM).

Month/parameter	Yolk diameter	Yolk colour	Yolk colour	Yolk colour	Yolk colour	Yolk pH	Albumen pH
	( cm )	with fan	$L^{*}$	$a^{*}$	$b^{*}$		
Jan ( n = 40)	7.09 ± 0.08^b^	12.58 ± 0.19^b^	66.48 ± 0.86	20.69 ± 2.83	54.57 ± 1.00^b^	6.18 ± 0.03^a^	8.73 ± 0.03
Feb ( n = 40)	6.59 ± 0.08^a^	11.83 ± 0.19^a^	63.86 ± 0.86	16.87 ± 2.83	49.91 ± 1.00^a^	6.29 ± 0.03^b^	8.70 ± 0.03
Large group ( n = 20)	6.91 ± 0.08	12.16 ± 0.19	65.45 ± 0.83	20.04 ± 2.74	52.66 ± 0.97	6.22 ± 0.03	8.67 ± 0.03^a^
Small groups ( n = 60)	6.77 ± 0.08	12.32 ± 0.20	65.04 ± 0.88	17.52 ± 2.91	51.82 ± 1.71	6.24 ± 0.03	8.77 ± 0.03^b^
Large group in Feb ( n = 10)	6.60 ± 0.09	11.67 ± 0.16	64.30 ± 1.09	15.97 ± 0.98	49.7 ± 1.22	6.24 ± 0.33	8.64 ± 0.04
Small groups in Feb ( n = 30)	6.58 ± 0.06	11.83 ± 0.09	64.63 ± 0.67	17.48 ± 0.59	49.44 ± 0.75	6.29 ± 0.20	8.67 ± 0.03

^a, b^ Letters indicate significant differences ( P ≤ 0.05).

Yolk diameter, yolk colour measured with a fan, and $b^{*}$ decreased, while yolk pH increased from January to February. Regarding the effect of group size, only albumen pH changed, showing a higher mean value in small groups (Table 5). The grand means of the examined yolk and albumen characteristics were the following: yolk diameter was 6.84 cm , yolk colour (fan) 12.21, $L^{*}$ 65.17, $a^{*}$ 18.78, $b^{*}$ 52.24, yolk pH 6.23, ad albumen pH 8.72. Yolk characteristics and albumen pH did not differ by groups in February ( P = 0.20 to 0.87).

Egg yolk colour is influenced by many factors, such as breed, nutrition, feed additives (oxy-carotenoids), and intensity of egg production (Karunajeewa et al., 1984). In the CIELAB colour measurement, $L^{*}$ defines the lightness of colour, $a^{*}$ determines the greenness and redness, and $b^{*}$ shows the blueness and yellowness of the investigated material and tissue (Hernández Salueña et al., 2019). Although Kim et al. (2015) found that the yolk colour of a laying hen is more intensive in the red plane due to dietary corticosterone supplementation, we could not reveal changes in $a^{*}$ . The mean albumen pH was strongly alkaline, with it being within the range (7.6–9.7) reported for poultry eggs (Sharp and Powell, 1931). More references including the mentioned parameters and effects are not available in either goose or other poultry species.

**Table 6:** The IgG and corticosterone concentration of egg yolks in the large and small groups (mean ± SEM).

Group	IgG ( $\mathrm{ng}\;{\mathrm{mL}}^{-1}$ )	Corticosterone ( $\mathrm{ng}\;{\mathrm{mL}}^{-1}$ )
Large group in Feb ( n = 8)	3823.17 ± 365.0^a^	76.24 ± 5.13^a^
Small groups in Feb ( n = 8)	4955.95 ± 352.5^b^	187.26 ± 28.64^b^

^a, b^ Letters indicate significant differences ( P ≤ 0.05).

### 3.2 The effect of group size on IgG and corticosterone levels

In order to monitor stress and immunity of goose eggs, IgG and corticosterone levels were measured by ELISA assays. IgG concentration in the small groups was significantly higher compared to the large group ( P = 0.049) (Table 6).

To the best of our knowledge, literature on the effect of group size on blood plasma, serum, or yolk IgG concentration is not available. Instead, results on high stocking density in calves and high stocking density and heat stress in ducks indicated that the serum IgG concentration was either unchanged or elevated due to the stressful environments (Fujiwara et al., 2020). In pigs, the IgG level was not influenced by the heat and crowding stress (Sutherland et al., 2006). On the contrary, El-Shafei et al. (2012) observed that IgG increased significantly ( P ≤ 0.05) due to decreasing stocking density, thus leading to increased susceptibility to resistance of infections. Hofmann et al. (2021) described that the plasma concentration of IgG and corticosterone was not affected by stocking density in laying hens. In our experiment, the population density of the small and large groups was the same at 1 $m^{2}\;\mathrm{per}\;\mathrm{bird}$ .

Corticosterone concentration in the small groups was significantly higher compared to the large group ( P = 0.0078). Conversely, the literature reports that in poultry and pigs, the plasma corticosterone concentration increased in larger group sizes (Rodenburg and Koene, 2007). A possible explanation for the higher corticosterone levels in the small group could be that the geese in the small groups stemmed from the original population with a large size. Early rearing conditions may be of particular importance for poultry to adapt to future housing systems involving large group sizes (Moe et al., 2010). In our case, the animals in the small groups had already adapted to the large group size in their earlier years; therefore, the reduction in the usual group size could have caused the elevated corticosterone levels. In our study, the higher corticosterone levels in the small groups were associated with higher IgG levels. In agreement with our results, Yang et al. (2015) determined that the corticosterone administration resulted in increased plasma IgG levels in broiler chickens. Even though there were significant differences between the small and large groups in both IgG and corticosterone levels during February, egg quality parameters did not differ in this respect. In agreement with our results, Kim et al. (2015) stated that the elevated corticosterone level did not affect egg weight and eggshell weight. However, our result on egg thickness contradicts their results on eggshell thickness since the authors found that higher corticosterone content results in thicker eggshells (Kim et al., 2015). Regarding the effect of stress on egg quality, heat stress is a more abundantly discussed topic in the literature. Melesse et al. (2010), for example, indicated a decrease in shell thickness due to heat stress. They also declared that heat stress negatively affects egg weight and specific gravity. Chen et al. (2023) stated that heat stress decreased yolk weight. A reference on the effect of stress or corticosterone on other egg parameters is not available.

## 4 Conclusions

Most changes in egg quality parameters were declared as the effect of the laying month. According to our results, except for albumen pH, group size did not affect egg quality parameters, while it had significant effects on the IgG, and corticosterone levels. So, the common change in egg quality, IgG and corticosterone levels due to the effect of group size could not be proven. Also, international reviews are too scarce and contradictory to come to further conclusions. For the future, it can be suggested to extend the scope of examinations to the number of eggs per bird and hatchability as affected variables to draw more precise conclusions. The effects of laying months and group sizes are obvious on egg characteristics; however, more eggs will need to be involved in the experiment to draw further conclusions on the stress and immune parameters of goose eggs.

## Data Availability

Data are available from the corresponding author upon reasonable request.
